# Computer-Aided Detection of Respiratory Sounds in Bronchial Asthma Patients Based on Machine Learning Method

**DOI:** 10.17691/stm2022.14.5.05

**Published:** 2022-09-29

**Authors:** A. Gelman, E.G. Furman, N.M. Kalinina, S.V. Malinin, G.B. Furman, V.S. Sheludko, V.L. Sokolovsky

**Affiliations:** Laboratory Engineer, Department of Physics; Ben-Gurion University of the Negev, P.O.B. 653, Beer-Sheva, 8410501, Israel;; Professor, Corresponding Member of Russian Academy of Sciences, Head of Faculty and Hospital Pediatrics Department; Perm State Medical University named after Academician E.A. Wagner, 26 Petropavlovskaya St., Perm, 614990, Russia; Resident; Perm State Medical University named after Academician E.A. Wagner, 26 Petropavlovskaya St., Perm, 614990, Russia; Researcher; Perm State Medical University named after Academician E.A. Wagner, 26 Petropavlovskaya St., Perm, 614990, Russia; Professor, Department of Physics; Ben-Gurion University of the Negev, P.O.B. 653, Beer-Sheva, 8410501, Israel;; Leading Researcher, Central Scientific Research Laboratory; Perm State Medical University named after Academician E.A. Wagner, 26 Petropavlovskaya St., Perm, 614990, Russia; Professor, Department of Physics; Ben-Gurion University of the Negev, P.O.B. 653, Beer-Sheva, 8410501, Israel;

**Keywords:** bronchial asthma, respiratory sounds, computer-aided diagnostics, machine learning, neural network

## Abstract

**The aim of the study** is to develop a method for detection of pathological respiratory sound, caused by bronchial asthma, with the aid of machine learning techniques.

**Materials and Methods:**

To build and train neural networks, we used the records of respiratory sounds of bronchial asthma patients at different stages of the disease (n=951) aged from several months to 47 years old and healthy volunteers (n=167). The sounds were recorded with calm breathing at four points: at the oral cavity, above the trachea, on the chest (second intercostal space on the right side), and at a point on the back.

**Results:**

The method developed for computer-aided detection of respiratory sounds allows to diagnose sounds typical for bronchial asthma in 89.4% of cases with 89.3% sensitivity and 86.0% specificity regardless of sex and age of the patients, stage of the disease, and the point of sound recording.

## Introduction

It is known that diagnosis of bronchial asthma (BA) is based on patient’s comprehensive dynamic examination including determination of the lung functional state [1]. One of the main tasks of asthma management is to early diagnose and to timely start the treatment along with achieving control of the patient’s condition to prevent exacerbations and occurrence of the disease severe forms, thus requiring continuous monitoring including remote out-of-hospital control [1]. Rapid and objective determination of prescribed medications effectiveness is of equal relevance. Studying the lung functional state may be difficult in some cases of pediatric practice (children of the preschool age do not interact sufficiently during such procedures). In addition, the results of physical examination by auscultation of the lungs are largely subjective. Sometimes such diagnosis is delayed, as is the case, in the COVID-19 pandemic [2, 3].

Computer-aided analysis of respiratory sounds may complement the screening diagnosis of pulmonary diseases including BA [4-8]. Computer-aided detection methods are able to analyze changes of respiratory sounds that cannot be detected by a human ear. Respiratory sounds accompanying bronchoobstructive syndrome are characterized by the occurrence/ amplification of a set of periodic waves in the frequency range of 100 to 2500 Hz. The basic frequency of these sounds is between 100 and 1000 Hz and between 400 and 1600 Hz, respectively [9, 10]. The dominant frequency of whistling breathing is above 400 Hz, while that of wet wheezing is about 200 Hz or lower [2, 10]. In patients with BA, whistling sounds are observed in the exhalation phase, with breathy whistle duration ranging from 80 to 250 ms. These features of respiratory sounds allowed to develop methods of computer-aided diagnostics of bronchoobstructive syndrome in children [4, 5, 11–13]. These methods are based on automated comparison of the Fourier spectra for respiratory sounds of healthy and asthmatic children and demonstrate the greater diagnostic values for BA (AUC varies from 0.783 to 0.895). Besides, the variability of this sound phenomenon cannot be excluded depending on individual characteristics of the patient (age, sex, stage of disease, physical development parameters).

The use of modern electronic devices (computers, cell phones, etc.), communication means and software allows developing new alternative methods of computer-aided diagnostics based on analysis of the patients’ breath sounds. These methods include machine learning and deep machine learning (deep learning) [8, 14, 15]. Recently, the possibility of applying these methods in various fields of medicine was actively studied [8, 14, 16–20]. Today, a large number of machine learning methods exist allowing to create algorithms for decision-making in this or that situation and suitable for different tasks, including medical ones: e.g. different types of cancer classification [17], analysis of dialysis efficiency [17], diagnosis of lung diseases [8, 14, 19, 21]. In particular, it is proposed to apply machine learning methods in pulmonology: to sort patients with chronic obstructive pulmonary disease [20], to automatically classify respiratory sound spectra of patients with lung diseases [22], to diagnose BA and chronic obstructive pulmonary disease [23], to rank clinical assessment of asthma in children [24].

The main idea of machine learning is to facilitate data processing, to highlight exactly the information that will be useful in the diagnosis, and to make a verdict (establish a diagnosis) on the basis of this information. Unlike methods based on detection of pathological changes (coughs, wheezes, sibilant rales, whistles, etc.) of breath sounds, machine learning methods analyze the most general characteristics of sounds (mean frequencies, frequency moments, etc.) and their variations caused by the disease. These methods are often less interpretable but demonstrate greater reliability of predictions [25-27]. In machine learning methods, the input objects are a set of patient characteristics, and the output is class number — diagnosis (response). Correlation exists between the input data and the output (diagnosis) which must be determined during program training. To establish this correlation, training samples, a large database containing a set of characteristics of people with a known diagnosis should be generated.

In this paper, machine learning methods were used to develop a computer-aided method for detecting pathological respiratory sounds occurring in BA patients to supplement the disease diagnosis with objective tools. Our proposed method is based on a comparison of respiratory sounds of patients with BA and healthy volunteers.

## Materials and Methods

The research design corresponded to a cross-sectional observational one-time sample study. Patients were clinically examined, and respiratory sounds were recorded at the Regional Children’s Clinical Hospital of Perm and the polyclinic of Perm State Medical University named after Academician E.A. Wagner (Russia). The research was performed in accordance with the Declaration of Helsinki (2013) and was approved by the local Ethics Committee. Written informed consent was obtained from each person (in the case of children, from their parents or guardians) in accordance with Federal Law No.54871 of July 22, 1993 “Principles of legislation of the Russian Federation on protection of citizens’ health”.

Bronchial asthma in children was diagnosed in accordance with the recommendations presented in the National Program “Bronchial asthma in children. Treatment strategy and prevention” [28], in adult patients — according to the recommendations of the Ministry of Health of the Russian Federation “Bronchial asthma. Clinical recommendations” [29].

### Database

The anonymous database, along with files containing digitized audio records of respiratory sounds, includes main characteristics of the persons: age, sex, information on state of health (healthy, exacerbation of BA or its remission), time and point of recording (see section “Respiratory sound recording”). The database includes 951 respiratory sound records of asthmatic patients aged from a few months to 47 years and 167 records of healthy volunteers in the same age range. Percentage distribution of entries by age: 0–2 years old — 1.6%; 2–4 — 0.7%; 4–13 — 43.8%; 13–19 — 42.5%, 20 and older — 11.4%. The records were distributed among men and women as 67 and 33% ratio, respectively. 232 records were made from patients in exacerbation, 309 in remission, and 410 in incomplete remission.

Auscultatory findings in patients with BA were characterized by harsh breathing, diffuse dry sibilant rales over both lungs. In healthy volunteers, vesicular breathing with good sound transmission to all parts of the lungs was heard over the lungs. Volunteers did not suffer from lung disease or other diseases causing pathological changes in respiratory sounds at the time of recording.

The database contains no sound records of adult patients with bronchial obstruction syndrome accompanied by other diseases.

### Respiratory sound recording

Respiratory sounds were recorded at four points during calm breathing: point 1 — in the oral cavity (4.3% of all records); point 2 — above the trachea (50.2%); point 3 — on the chest, second intercostal space on the right side (23.6%); point 4 — on the back (21.9%). In the majority of the exam persons, the records were made at several points, for some patients — at different stages of the disease (exacerbation, incomplete remission, and remission). Recording systems and quality of the records met the requirements we formulated earlier [11]. Record quality was controlled visually (record oscillograms were displayed on the computer screen) and using a developed program.

Breath sounds were recorded continuously for several respiratory cycles (approximately 25 s). This reduced the influence of sound intensity random variations on the results.

Respiratory sounds were recorded using cell phones and computer systems. Files recorded on phones using the built-in and/or external microphones were sent to a “cloud” to create an anonymous database. Computer-aided records were made using systems for recording respiratory sounds [4, 5, 7, 11] with external microphones, electronic phonendoscopes, and computer sound cards; with the Adobe Audition audio editor. All systems exhibited high amplitude-frequency linearity in the 100 to 3000 Hz frequency range. The sampling rate was varied from 22 to 96 kHz.

### Method of data processing

The authors of papers [8, 21, 30] discuss a variety of machine learning methods that can be used for development of computer-aided diagnostics.

In our research, we defined two categories: “sick” and “healthy”. In this regard, the most suitable approach for diagnosing pathological sounds is using the Sequential Neural Network [31].

The proposed method is based on the following: the audio record of respiratory sounds is converted into a time series with characteristics that can be investigated by any method suitable for time series analysis. These characteristics were used to train the neural network and develop the express analysis methods. Visually, a respiratory sound Fourier spectrum of asthmatic patients contains areas of frequencies where amplitudes of harmonics significantly exceed the amplitudes of a healthy patients’ spectrum (see e.g., Figure 4 in [4] and Figure 3 in [2]). We observed a periodic manifestation of such amplification lasting about 200 ms (see e.g., Figure 5 in [4]). Note that asthmatic disease is often accompanied by respiratory rate increase. These differences between respiratory sound characteristics of a patient and a healthy person allowed us to choose the following parameters for building the neural network training: spectral bandwidth, spectral centroid, zero-crossing rate, spectral roll-off, and chroma feature [32].

Spectral width was measured at spectrum half maximum intensity, and it shows how much energy is concentrated or dispersed as a function of frequency.

The spectral centroid illustrates the position of the spectrum energy center and is defined as

fc=∑kS(k)fk∑kS(k),

where *S*(*k*) is the amplitude of the *k*^_th_^ harmonics with the *f_k_* frequency.

The number of the zero crossings describes the sound record in terms of high-frequency harmonics, mathematically showing the smoothness of the signal.

Spectral decay is a waveform measure representing the frequency at which amplitudes of high-frequency harmonics decrease to zero.

Chroma feature is a vector usually consisting of 12 elements. To determine vector elements, the spectrum was divided into 12 sections (classes) and the element was determined by the amount of energy in the class. Chroma feature was used to describe the measure of similarity between signals.

For fast calculation of the above noted parameters, built-in functions of the Python Librosa library were used (chroma_stft, spectral_centroid, spectral_bandwidth, spectral_rolloff, zero_crossing_rate). Respiratory sound records contain up to a million points, so using other software can lead to a significant calculation time increase. In the standard library, these functions are optimized and operate fast. After calculating the parameters, their values were centered and normalized, as this representation of the data is better suited for machine learning methods.

**Figure 1.**
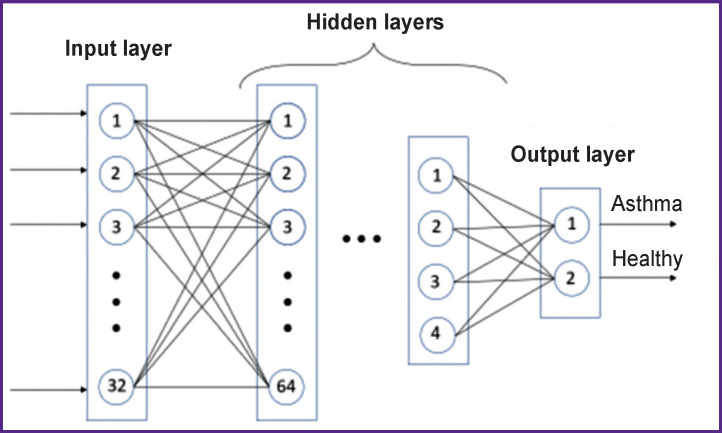
Simplified diagram of the neural network

A program in Python (version 3.8) using Keras and Librosa libraries was developed to process the audio files. The first library serves to build a neural network, the second one is used for simple and fast analysis of audio files. Analysis of an audio file requires about one second. A sequential model from Keras library was chosen to build the neural network. It allows to create a network of multiple layers, to train the network and to define a predicted category (category classification), which the object should belong to (its characteristics are fed to the input). A neural network should consist of at least two layers of so-called neurons being functions that process the analyzed object parameters and activate the next layer element depending on the previous layer result [33-37]. The neural network we developed contains 10 layers. A signal characteristics vector consisting of 32 elements is fed to the input. In addition to the above signal parameters, the so-called mel-frequency cepstral coefficients were also used being widely applied in analysis of audio signals and in speech recognition tasks as they allow improving classification quality of breath sounds. 32 network elements are introduced into the input layer, 2 ones are obtained at the output layer, and there are 10 hidden (inner) layers between these two layers, with elements number first increasing to 128 (according to degrees of number 2) and then decreasing to 2 at the output. As the problem solution, we mean a binary classification “asthma/healthy”; the algorithm determines at the output the probability of the input data belonging to one of the two groups. If the algorithm results in a probability of any group greater than 50%, it is assumed that the input data belong to that group. Consequently, the sum of probabilities for the two groups equals 1. Figure 1 shows a simplified diagram of the neural network. More detailed description of designing such circuits is presented in [38]. For each layer, except for the output layer, a rectified linear unit (ReLU) activation function was used, which is the most commonly used activation function in deep learning. This function equals zero for argument negative values and equals the argument for its positive values. At the output layer, the activation function, softmax, was used, which creates a probability distribution and determines the probability of matching the input data to one of the classes.

The database of respiratory sounds records was divided into two groups:

the first, a training one (with 374 records of asthmatic patients’ respiratory sounds and 146 records of healthy patients) was used to train the network;

the second, a control one (577 patients and 21 healthy volunteers) was used to check the network operation and correctness of computer-aided diagnostics of sounds.

In these groups, the persons (their records) were almost alike distributed by age, sex, and recording points. None of the records included in the first group were used in the second (control) group. The implemented division was dictated, on the one hand, by the need to “balance” the training group (coincidence was desirable of the number of records of patients and healthy people); on the other hand, the number of records used to train the network must be large (several hundred at least). In this research, patients were not classified by stages of disease when training and testing the network. To demonstrate the universality of the approach, the training group contained data recorded using various devices at different recording speeds of 44 to 96 kHz, and the control group — only computer records at 22 kHz.

The first group was divided into the testing and training subgroups arranged in a 20/80 ratio by random selection. The training consisted of 100 epochs. Numerical experiments on different subgroups showed that this number of epochs was sufficient to train the model: the diagnostic accuracy reached an asymptotic value. Diagnostic accuracy for the testing subgroups reached 90% for patients and 87% for healthy volunteers and varied within ±1% when the testing and training subgroups were altered.

Computer-aided analysis of respiratory sounds was carried out at Ben-Gurion University (Israel) using a developed software based on Python scripting language. Tkinter library was used as a user-friendly graphical interface (GUI).

## Results

The developed program was used to analyze the respiratory sounds of the second (control) group, none of its records were included in the first group.

The developed program correctly diagnosed sounds as belonging to a patient with BA in 516 cases out of 577 records (89.4%); in 55 cases (9.5%), records were incorrectly identified as made in healthy individuals, and in 6 cases (1.1%), the result was not uniquely determined. Among 21 records made in healthy volunteers, in 18 cases (85.7%) the program correctly identified the records as made in volunteers. The obtained results allow us to assess statistical metrics:

1. Categorical accuracy was 89.4% (asthma); 85.7% (healthy).
2. The method sensitivity was 89.3% with 86.0% specificity.
3. The test accuracy was determined by the formula:

A=Tp+ThTp+Fp+Th+Fh100%=89.1%,

where *T_p_* and *T_h_* are the numbers of correctly diagnosed patients and healthy volunteers, respectively; *F_p_* and *F_p_* are the numbers of misdiagnosed patients as healthy and of volunteers as sick, respectively.

The test accuracy in our case is almost identical to the sensitivity. This can be explained by a strong “imbalance” in the number of records for sick and healthy people: the number of records for patients exceeds by ~30 times those for volunteers. Approximation to the case of a “balanced” control group (the numbers of records for sick and healthy people being the same) allows us to achieve the accuracy as 87.5%.

Youden’s index was calculated according to the formula:

J=(TpTp+Fp+ThTh+Fh−1)=0.753.

Youden’s index shows the probability of taking an informed decision given all projections. Youden’s index value of 0.753 indicates a sufficiently high reliability of the diagnostic approach we developed.

Accuracy value of 85.7% for sound diagnosis as healthy patients’ sounds is not representative, as the control group contained a small number of records of healthy volunteers (n=21). However, high accuracy (89.4%) of asthma patients’ sound classification in the control group and in the testing subgroups (90±1% for patients and 87±1% for healthy persons) allows stating that the developed approach and trained model provide diagnosis (classification) of respiratory sounds with high reliability. Accuracy improvement can be achieved via increasing the database and, first of all, the number of volunteer respiratory sound records. This will increase the diagnostic accuracy of the method by creating trained programs for different age groups and separating patients by sex.

## Discussion

**Figure 2.**
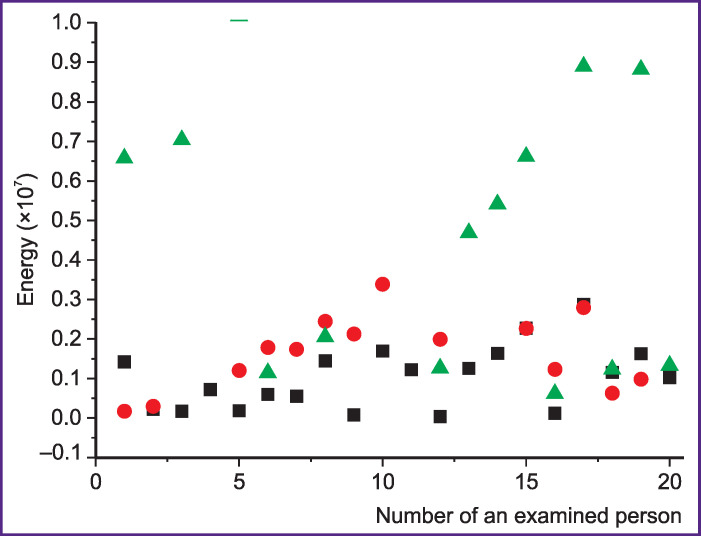
Respiratory sound energy: black squares — for healthy people; red circles — for asthmatic patients in the exacerbation stage; green triangles — for asthmatic patients in remission. The sound energy determined by the method proposed in [5] is given in arbitrary units (the vertical axis). The numbering (the horizontal axis) of healthy volunteers and patients in both stages is independent. As example, the results for 20 persons in each group are shown

The research results showed that the selected parameters are sufficient for the design and training of the network, as their use allows achieving a high level of reliability of computer-aided diagnostics. These parameters are independent of the person’s age, sex, and of the recording point. They can be used for BA diagnosis at different stages (exacerbation, remission, incomplete remission). BA is characterized by obstruction and inflammation affecting the entire airway from central to peripheral areas of the tracheobronchial tree (small bronchi) [39-41]. These pathological changes in the respiratory tract lead to changes in respiratory sounds, i.e. changes in the amplitude-frequency characteristic of sounds (change in the Fourier spectrum, surge of additional harmonics). The use of medications reduces inflammation and dilates patient’s airways in the partial remission or remission stage. Although external signs of the disease (labored breathing, shortness of breath, rapid breathing) are not always observed at these stages, lung function is not fully restored, this being reflected in the characteristics of respiratory sounds. Moreover, manifestations of pathological processes in respiratory sounds of patients in the remission stage are more pronounced than for patients during exacerbation. Thus, pathological respiratory sound energy in the remission stage can be greater than during exacerbation (Figure 2).

The achieved characteristics of the developed method (89.3% sensitivity and 86.0% specificity) exceed those of traditional diagnostic methods (e.g., spirometry). Thus, sensitivity in research [42] was 29% (when spirometry is carried out by high-level specialists and the patient cooperates actively, 39.8% may be reached) with 90% specificity; when diagnosing the disease in children [43], sensitivity was 31% with 90% specificity; changing peak expiratory rate in SAPALDIA research [44] gave 40% sensitivity with 83% specificity.

High reliability of asthmatic respiratory sounds detection allows using the developed method and the trained program as an addition to medical auscultation for BA diagnosis. Unlike lung auscultation carried out by a physician, the results of computer-aided diagnostics are independent of any subjective assessment and have a higher sensitivity when the level of pathological sounds is low and cannot be discerned by the human ear against the background of other noises.

The developed method can be used as an additional express method for screening-diagnostics of bronchial asthma and serve as a basis for the development of methods for computer-aided control over the patient’s condition and efficiency of medication administration in real time. The proposed method can be used to diagnose and monitor the health of children under 5 years of age to whom it is difficult to apply physical examination and spirometry; to diagnose patients from remote areas; for rapid diagnosis of patients outside the hospital; to find application in telemedicine.

## Conclusion

The developed method of computer-aided diagnostics based on machine learning methods allows one to classify (diagnose) respiratory sounds as sounds of a patient with bronchial asthma with high reliability: 89.3% sensitivity; 86.0% specificity; ~88.0% accuracy and Youden’s index — 0.753.
